# Reducing surgical levels by paraspinal mapping and diffusion tensor imaging techniques in lumbar spinal stenosis

**DOI:** 10.1186/s13018-016-0382-1

**Published:** 2016-04-25

**Authors:** Hua-Biao Chen, Qi Wan, Qi-Feng Xu, Yi Chen, Bo Bai

**Affiliations:** Department of Orthopaedic, First Affiliated Hospital, Guangzhou Medical University, 151 Yanjiang Road, Guangzhou, 510120 People’s Republic of China; Department of Radiology, First Affiliated Hospital, Guangzhou Medical University, 151 Yanjiang Road, Guangzhou, 510120 People’s Republic of China; Department of Electromyography, First Affiliated Hospital, Guangzhou Medical University, 151 Yanjiang Road, Guangzhou, 510120 People’s Republic of China; Guangdong Key Laboratory of Orthopaedic Technology and Implant Materials, First Affiliated Hospital, Guangzhou Medical University, 151 Yanjiang Road, Guangzhou, 510120 People’s Republic of China

**Keywords:** Diffuse tensor imagining, Lumbar spinal stenosis, Oswestry Disability Index, Paraspinal mapping, Visual analog pain scale

## Abstract

**Background:**

Correlating symptoms and physical examination findings with surgical levels based on common imaging results is not reliable. In patients who have no concordance between radiological and clinical symptoms, the surgical levels determined by conventional magnetic resonance imaging (MRI) and neurogenic examination (NE) may lead to a more extensive surgery and significant complications. We aimed to confirm that whether the use of diffusion tensor imaging (DTI) and paraspinal mapping (PM) techniques can further prevent the occurrence of false positives with conventional MRI, distinguish which are clinically relevant from levels of cauda equina and/or nerve root lesions based on MRI, and determine and reduce the decompression levels of lumbar spinal stenosis than MRI + NE, while ensuring or improving surgical outcomes.

**Methods:**

We compared the data between patients who underwent MRI + (PM or DTI) and patients who underwent conventional MRI + NE to determine levels of decompression for the treatment of lumbar spinal stenosis. Outcome measures were assessed at 2 weeks, 3 months, 6 months, and 12 months postoperatively.

**Results:**

One hundred fourteen patients (59 in the control group, 54 in the experimental group) underwent decompression. The levels of decompression determined by MRI + (PM or DTI) in the experimental group were significantly less than that determined by MRI + NE in the control group (*p* = 0.000). The surgical time, blood loss, and surgical transfusion were significantly less in the experimental group (*p* = 0.001, *p* = 0.011, *p* = 0.001, respectively). There were no differences in improvement of the visual analog scale back and leg pain (VAS-BP, VAS-LP) scores and Oswestry Disability Index (ODI) scores at 2 weeks, 3 months, 6 months, and 12 months after operation between the experimental and control groups.

**Conclusions:**

MRI + (PM or DTI) showed clear benefits in determining decompression levels of lumbar spinal stenosis than MRI + NE. In patients with lumbar spinal stenosis, the use of PM and DTI techniques reduces decompression levels and increases safety and benefits of surgery.

## Background

The term lumbar spinal stenosis (LSS) is commonly used to describe patients with symptoms related to anatomical reduction in lumbar spinal canal. Among older individuals, LSS is a highly disabling condition [1] and is the most common reason for spinal surgery [2, 3]. The most common procedure involves a decompressive laminectomy of the structures thought to be causing nerve root irritation.

The challenge to the anatomically based determination is that while necessary for the diagnosis of LSS, it is not sufficient to determine the severity of symptoms that leads a patient to seek treatment [4]. The extent of narrowing of the spinal canal correlates poorly with symptom severity, and radiologically significant lumbar spinal stenosis can be found in asymptomatic individuals [4–7]. As a consequence, correlating symptoms and physical examination findings with decompression levels based on common imaging results is not reliable. In patients who have no concordance between radiological and clinical symptoms, the surgical levels determined by conventional magnetic resonance imaging (MRI) and neurogenic examination (NE) may lead to a more extensive surgery and significant complications. It is important to avoid inadequacies of MRI (MRI cannot precisely determine the lesion levels of lumbar spinal stenosis) in clinical practice.

Diffusion tensor imaging (DTI) is more sensitive than conventional MRI for precise determining the extent of spinal disorders via non-invasive, longitudinal examinations, in both humans and animal models. Moreover, the analysis of the fractional anisotropy (FA) proves more useful than other diffusional indices because of its simplicity, accuracy, and ability to reveal diverse spinal cord disorders, especially in clinical situations [8]. The quantification of the nerve root using the proposed methodology of DTI can identify the specific site of any degenerative and inflammatory changes along the nerve roots of patients with lower back pain [9].

Paraspinal mapping (PM) is a technique for needle electromyography (EMG) of the paraspinal muscles that has been the subject of several studies [10–12]. Although conventional imaging studies have a high false positive rate (a level with anatomical stenosis that is clinically irrelevant) for disc herniations, PM rarely produces evidence of radiculopathy in individuals without pain [12]. Theoretically, a single insertion into the location of each root level would assess for lesion in each root [13]. The PM is a sensitive method in the diagnosis of lumbar spinal stenosis and reflects physiology of the nerve roots better than the limb EMG [14]. Therefore, in the lumbar spinal stenosis, changing of DTI parameters (FA) or PM scores may possibly reflect the lesions of the cauda equina and/or spinal nerve roots more accurately than conventional MRI.

We hypothesized that the use of DTI and PM techniques can further prevent the occurrence of false positives with conventional MRI, distinguish which are clinically relevant from levels of cauda equina and/or nerve root lesions based on MRI, and determine and reduce the decompression levels of lumbar spinal stenosis than MRI + NE, while ensuring or improving surgical outcomes.

## Methods

### Enrollment and grouping

We enrolled symptomatic patients 20–90 years of age with degenerative lumbar spinal stenosis detected on magnetic resonance imaging (MRI) or radiography from October 2013 to October 2015 at Orthopedics of First Affiliated Hospital of Guangzhou Medical University. Since stenosis-defining features can be seen on MRI before and clearer than changes consistent with stenosis can be detected on radiography, patients with degenerative lumbar spinal stenosis on MRI were eligible. We required that patients had neuroclaudication with lower back pain and one leg pain that was consistent with a lumbar spinal stenosis and had persisted for at least 1 month despite pharmacologic treatment, physical therapy, or limitation of activity. Leg pain was defined as pain below the buttocks [15]. Neurogenic claudication was typical with severe pain and/or disability and a pronounced constriction of the lumbar spinal canal; therefore, it was considered for decompression treatment [16]. Neurogenic examination (NE) was performed by an experienced spine surgeon; he was blinded to the treatment of patients. Levels of decompression determined only by MRI were ≥2. Patients were excluded if they had diabetes, history of heavy alcohol consumption, history of lower back surgery [17, 18], evidence of polyneuropathy, or technically inadequate MRI or EMG results.

All patients were randomly chosen by tossing a coin to DTI and PM examinations, the ones who underwent DTI and PM examinations were the experimental group and the others were the control group. Tossing a coin was performed by a trained spine surgeon who was blinded to the treatment of patients.

### Interventions

Patients went for decompression surgery with decompression levels determined by MRI + (PM or DTI) in the experimental group while by MRI + NE in the control group. All surgeons were trained and performed at least 50 lumbar spinal decompression surgeries annually. PM and DTI were described below.

### Paraspinal mapping (MiniPM)

**Fig. 1:**
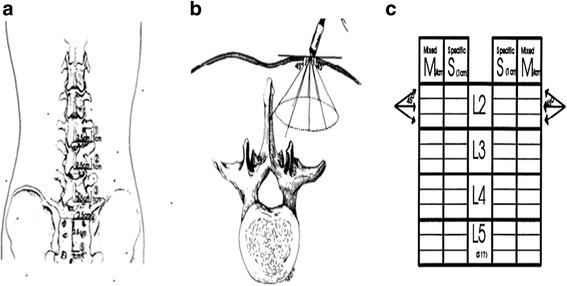
**a** With the patient prone, palpate 2.5 cm lateral and 1.0 cm cranial to the inferior edge of the spinous processes of L3, L4, and L5 and measure L2, L3, and L4 nerve roots, respectively. Mark a fourth location 2.5 cm lateral to the midline between the tips of the posterior superior iliac spines. Mark a fifth location 2.5 cm down to the midpoint and 1.0 cm lateral to the midline between the tips of the posterior superior iliac spines [20]. In this study, palpate 2.5 cm lateral and 1.0 cm cranial to the inferior edge of the spinous processes of L2 which was added to measure the L1 nerve root [21]. **b** Directions of needle insertion at each location. On the medial three insertions, the final l cm before contacting midline is scored “S” for specific. The remainder of these three insertions are scored “M” for medial. Note that the upper and lower medial insertions may not hit the spinous process before the needle hub touches skin, while the central medial insertion should do so if palpation was correct [20]. **c** The scoresheet: spontaneous activity is scored separately for insertions within the first 4 cm of insertion (placed in the M column on the scoresheet) and in the last l cm of insertion (placed in the S column of the scoresheet) [13]. In this study, L1 and S1 nerve roots were added to the scoresheet. PM scores were the summary of all plus at one nerve root level at one side

The technique for MiniPM has been described in detail elsewhere [13]. Briefly, as shown in Fig. 1a, b, four locations on the most symptomatic side of the lumbar area are palpated. A 50- or 75-mm monopolar EMG needle is inserted into each of these locations and directed toward the midline, cranial medial, and caudal medial. For each of these 12 insertions, the medial-most 1 cm is scored separately from the lateral part of the insertion [19]. At skin puncture site 5, there are only 9 scores, because position 5 has no medial insertions more than 1 cm from the midline [20]. Abnormalities are coded 0 to 4+ in each of these 24 locations, and a total MiniPM score is summed by totalling all the pluses.

**Fig. 2:**
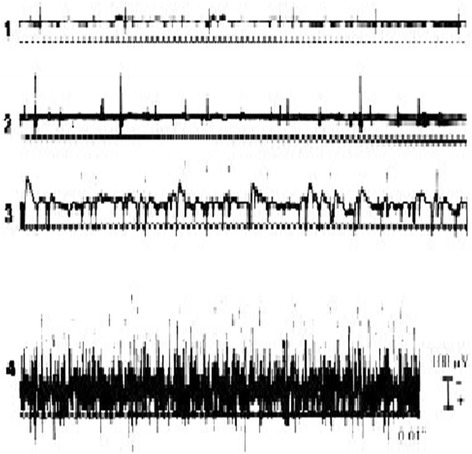
Fibrillation potentials in denervated muscle. Grades of activity: 1+, fibrillation potentials persistent in at least two areas; 2+, moderate number of persistent fibrillation potentials in three or more areas; 3+, large number of persistent discharges in all areas; 4+, profuse, widespread, persistent discharges that fill the baseline [53]

Typically, the total MiniPM score is used to indicate the extent of paraspinal denervation. In this study, the MiniPM score at each nerve root level, a summary of six scores (only a summary of three scores of the fifth needle point [S1 nerve root]) which shown to be associated with the neurologic level of a radiculopathy was used [21] (Fig. 1c). Denervation appeared if the paraspinal muscles showed fibrillation potentials, positive sharp waves, or complex repetitive discharges [22] (Fig. 2). Normal values established in 35 asymptomatic subjects are 0–2 (95 % scored <2), with a mean of 0.5 [12]; our pre-experiment also showed that if the PM scores of the level was ≥2 at one side, it was clinically meaningful and the level should be treated surgically. So we set the standard as follows: If a summary of six scores of one level (only a summary of three scores of the fifth needle point [S1 nerve root]) was ≥2 at one side, the level should be treated surgically. The PM examination was performed by a qualified electro-diagnostic physician who was blinded to the treatment of patients.

### MRI protocol

A 3.0 T MRI scanner (Achiva; Philips, Netherlands) was used in this study. Sagittal T1-weighted fast spin-echo sequences were obtained using a 453/8.0 ms for TR/TE, 4/0.4-mm section thickness/gap; 176 × 290 matrix; 0.91 × 1.00 × 4.00 mm^3^ actual voxel size; 0.50 × 0.50 × 4.00 mm^3^ calculated voxel size and sagittal T2-weighted fast spin-echo (TR/TE, 3604/110) sequences were obtained using a 4/0.4-mm section thickness/gap; 176 × 290 matrix; 0.91 × 1.00 × 4.00 mm^3^ actual voxel size and 0.50 × 0.50 × 4.00 mm^3^ calculated voxel size.

The quantitative criteria used for central anatomical LSS were as follows: The dural sac cross-sectional area (DSCSA) ≥100 mm^2^ was considered normal; 76 to 100 mm^2^ was considered to be moderately stenotic and ≤76 mm^2^ was considered as severely stenotic. Nerve root compromise in the lateral recess was graded as follows: grade 0, no contact of the disc with the nerve root; grade 1, contact without deviation; grade 2, nerve root deviation; grade 3, nerve root compression. Nerve root compression was considered to be present when the root was deformed [23]. The criteria for foraminal qualitative assessment were as follows: grade 0, normal foramina with normal dorsolateral border of the intervertebral disc and normal form of the foraminal epidural fat (oval or inverted pear shape); grade 1, slight foraminal stenosis and deformity of the epidural fat with the remaining fat still completely surrounding the exiting nerve root; grade 2, marked foraminal stenosis and deformity of the epidural fat with the remaining fat only partially surrounding the exiting nerve root; and grade 3, advanced stenosis with obliteration of the epidural fat [23, 24]. All the above were performed by a trained radiologist who was blinded to the treatment of patients.

#### DTI protocol

A 3 T MRI scanner (Achiva; Philips, Netherlands) was used in this study. Subjects were scanned in a supine position using an eight-channel phased array spine coil. DTI was performed using an echo-planar imaging sequence with a free-breathing scanning technique. The following imaging parameters were set: 0.600 s/mm^2^*b* value; MPG, 15 directions (Philips DTI medium); 6000/76 ms for TR/TE, respectively; axial section orientation, 3/0-mm section thickness/gap; 200 × 200 × 160 mm^3^ FOV; 64 × 78 matrix; 3.13 × 2.54 × 3.00 mm^3^ actual voxel size; 1.56 × 1.56 × 3.00 mm^3^ calculated voxel size; NSA, 3; 40 total sections; and 5 min 32 sec scan time.

T2-weighted 3D fast field echo sequence was obtained using a 33/3.9 ms for TR/TE; 80 × 80 matrix; FOV 160 × 160 × 200 mm^3^; NSA, 1; gap, 0 mm; 2.00 × 1.99 × 4.00 mm^3^ actual voxel size and 0.50 × 0.50 × 2.00 mm^3^ calculated voxel size.

### Image analysis

**Fig. 3:**
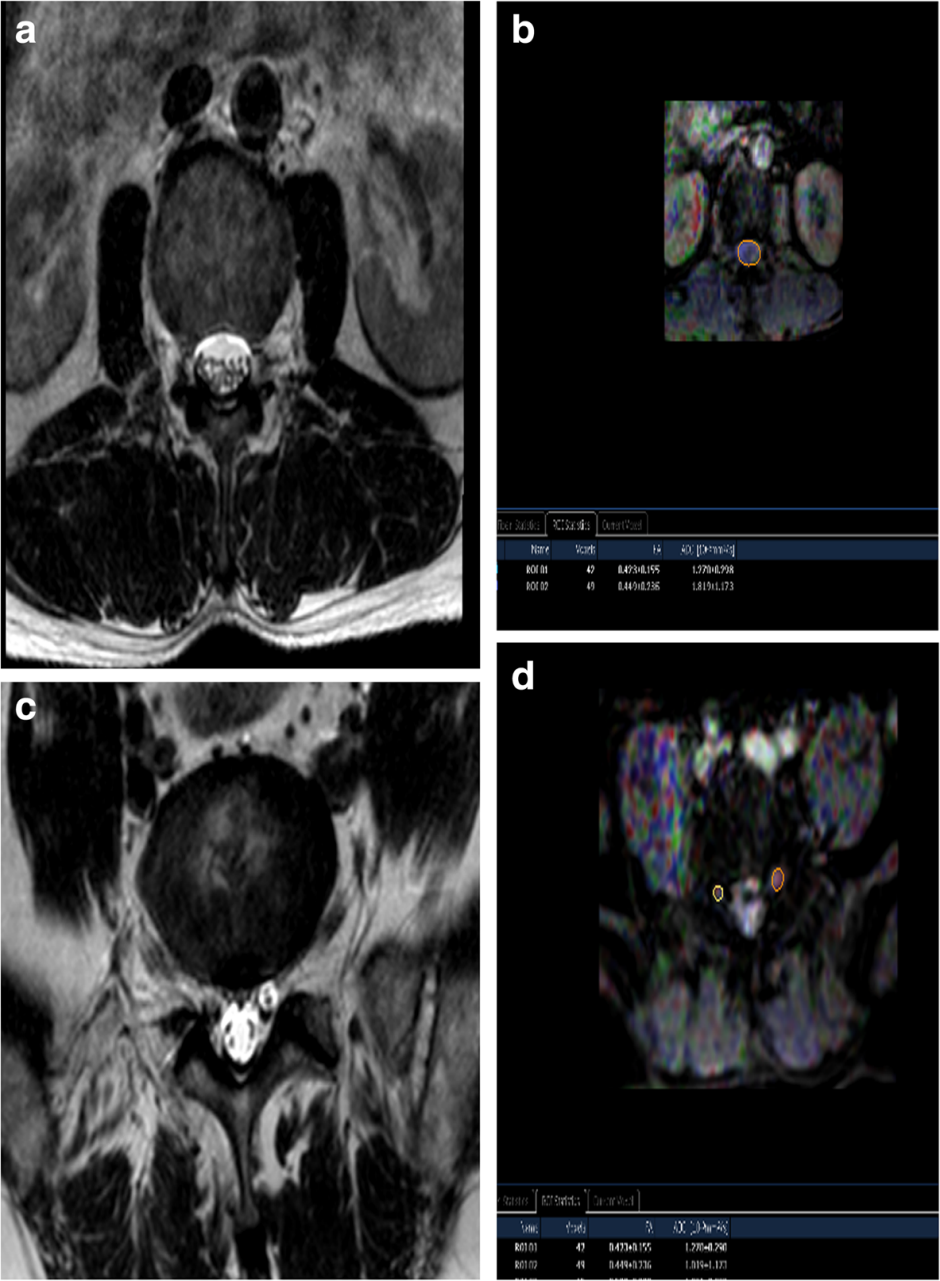
MRI T2W image of cauda equina (**a**) and FA mapping of DTI of cauda equina (**b**). ROIs were placed on the cauda equina on the zones equally as the disc, including superior 1/3, middle 1/3, and inferior 1/3 of the disc on FA mapping and FA values were calculated (**b**). The minimum values of three zones were taken as FA values of the cauda equina; MRI T2W image of bilateral nerve roots (**c**) and FA mapping of DTI of bilateral nerve roots (**d**). ROIs were placed on the “intraspinal,” “intraforaminal,” and “extraforaminal” zones of bilateral nerve roots on FA mapping and FA values were calculated (**d**). The minimum values of three zones were taken as FA values of the nerve roots. *MRI* magnetic resonance imaging, *FA* fractional anisotropy, *DTI* diffusion tensor imaging

**Fig. 4:**
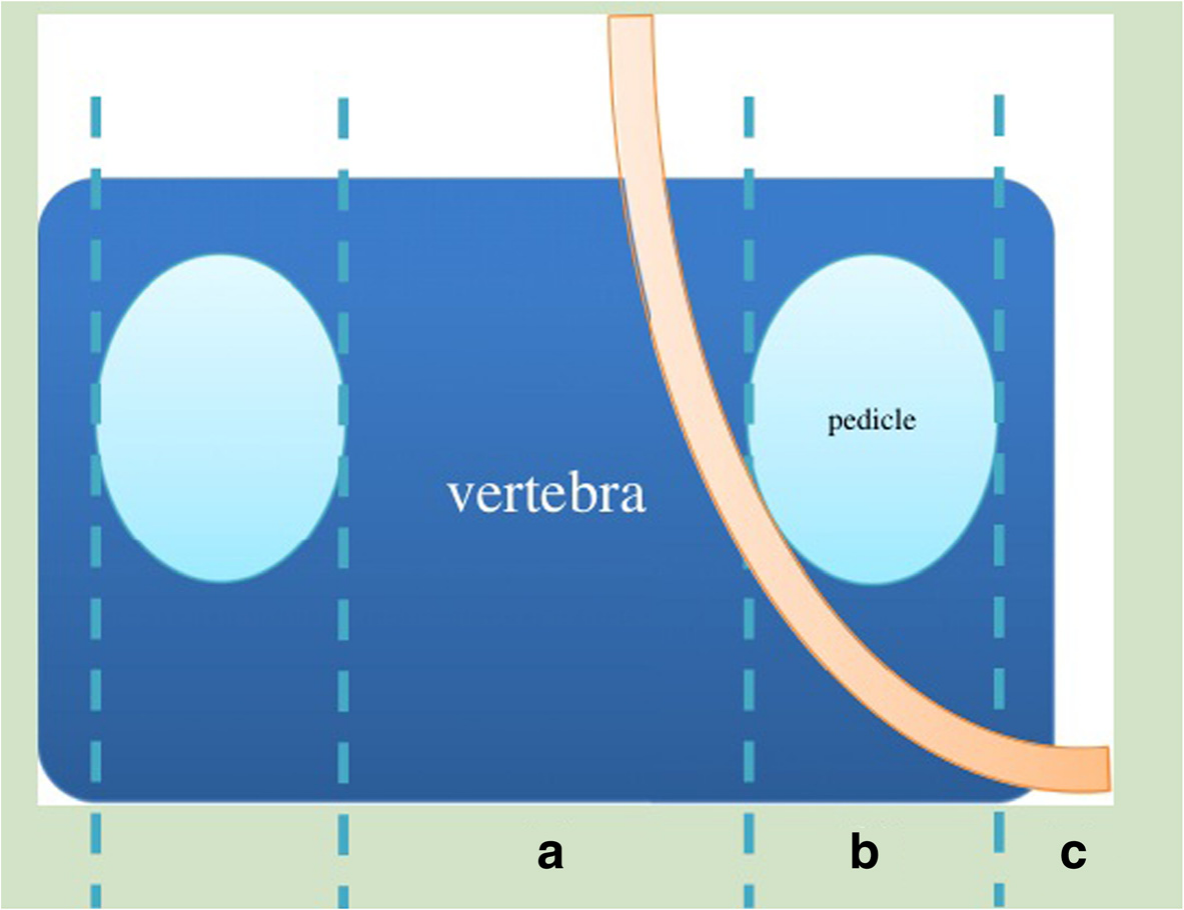
Zone definition of the spinal nerve root at the lumbar spinal canal. The area between the inner edge of both pedicles was defined as the intraspinal zone (**a**), the width of pedicle was defined as the intraforaminal zone (**b**), and the area outer to the outer edge of pedicle was defined as the extraforaminal zone (**c**) [25]

After DTI data were transferred to a PC, a Philips Extended Workspace (Philips DICOM Viewer R2.6 SP1) was used. Using the fiber tracking application software, anatomical images were superimposed on an FA map to permit the anatomical correlation (Fig. 3). The diffusion tensor was calculated using a log-linear fitting method. On axial images, the regions of interest (ROIs) were placed at cauda equina and the nerve roots of the level freehand, to circumscribe cauda equina and nerve roots with minimal inclusion of cerebral spinal fluid (CSF). In the cauda equina, ROIs were placed on the zones equally as the disc, including superior 1/3, middle 1/3, and inferior 1/3 of the disc, taking the minimum value of three zones as the FA value of the cauda equina. In lumbar spinal nerves, ROIs were placed on the “intraspinal,” “intraforaminal,” and “extraforaminal” zones (Fig. 4) [25], taking the minimum value of three zones as the FA value of the nerve root. FA values were calculated with the software at the levels of cauda equina and nerve roots from L1 to S1 in patients. The sizes of ROIs from 25 to 50 mm^2^ and 50 to 150 mm^2^ were selected to be as accurate as possible on the respective nerve roots and cauda equina to reduce the partial volume effects when the mean FA value was calculated. All DTI analyses were performed twice by two trained radiologists to avoid intra- and interobserver differences [26]; they were blinded to the treatment of patients. We set the standard as follows: If the FA value of lumbar cauda equina and/or nerve roots of the narrow level decreased ≥0.1 than that of the non-stenotic and normal level (commonly taken T12–L1 cauda equina and nerve roots value as reference), it was meaningful and the level should be treated surgically.

### Determining decompression levels

#### Determined by MRI + NE (control group)

A pronounced constriction of the lumbar spinal canal was considered the most important indication for surgical treatment [16]. The mean cross-sectional area of the dural tube at the narrowest level was 68.9 ± 25.7 mm^2^ in 47 patients with central stenosis [27] and based on the experience of our spine specialists that decompression levels generally were within the scope of the central tube ≤76 mm^2^ and/or foramen and/or lateral recess ≥grade 1 narrow. We set the standard as follows:

Levels of decompression include the level of the central tube ≤76 mm^2^ and/or foramen and/or lateral recess ≥grade 1 narrow determined by MRI and that located by NE in terms of the American Association of Spinal Cord Injury (ASIA). If NE cannot locate the level, it can be determined by MRI only.

#### Determined by MRI + (PM or DTI) [experimental group]

Based on MRI, the central tube ≤76 mm^2^ and/or foramen and/or lateral recess ≥grade 1 narrow, if the score of PM and/or the FA value of DTI was positive, the level was considered for surgical decompression; if the scores of PM and the FA value of DTI were both negative, the level was considered for surgical decompression determined by MRI only. If there was opinion conflict, the two direct of spine surgeons reached a mutual decision through discussion.

### Outcomes

#### In the experimental group

The primary outcomes were averages of reference FA values, positive, negative FA values, and positive, negative PM scores as well as the number of levels. All lumbar spinal levels were with respect to decompression levels determined only by MRI except for the reference levels. The reference FA value often referred to the FA value of L1, the positive and negative values of FA referred to the FA values of positive and negative levels, respectively, while the negative FA values of positive levels were excluded, each level including the cauda equina and nerve roots at the two sides. The negative and positive PM scores were PM scores of positive and negative levels, respectively; the negative PM scores in positive levels and levels of the PM scores = 0 were excluded, each level including nerve roots at the two side. The positive, specificity, and positive and negative predictive values of PM, DTI, and (PM or DTI) in distinguishing which are clinically relevant from the decompression levels were determined only by MRI. The levels of decompression were determined by MRI+ (PM or DTI) and MRI. Levels were clinically irrelevant and its percentage in that determined by MRI.

#### In the experimental and the control group

The primary outcomes were patient demographics, comorbidities, and surgical details. Comorbidities were classified as pulmonary, cardiac, metabolic, miscellaneous, and presence of spondylolisthesis or scoliosis [28, 29]. Surgical details include surgical time, blood loss, need for transfusion, and postoperative complications. All the above were performed by a trained spine surgeon who was blinded to the experiment of this study. Surgical decompression levels which were determined by MRI+ NE.

The secondary outcomes were the visual analog scale pain scores for both back and leg symptoms (VAS-BP, VAS-LP) and the Oswestry Disability Index (ODI), all of which have been used frequently in studies involving patients with lumbar spinal stenosis on a scale of 0 to 100 [28]. All patients were blinded to the role of pain scores and ODI.

#### Assessments

The primary outcomes were assessed at the preoperative stage. The secondary outcome was assessed at the preoperative stage and 2 weeks, 3 months, 6 months, and 12 months after surgery. Postoperative assessments were used to capture the trajectory and stability of the treatment response. Institutional ethics review board approval was obtained before commencing the collection of data.

#### Statistical analysis

All the measurement variable values were expressed as mean ± standard deviation.

#### In the experimental group

The primary analysis was implemented with an analysis of the covariance in FA values of positive levels and reference FA values, positive PM scores, and negative PM scores. *t* test analysis was undertaken to compare FA values of positive levels and reference FA values, positive PM scores, and negative PM scores. The positive, specificity, and positive and negative predictive value of PM, DTI, and (PM or DTI) in distinguishing which are clinically relevant from the decompression levels determined only by MRI were performed by diagnostic test.

#### In the experimental and the control group

The primary analysis was implemented with an analysis of the covariance in the decompression levels determined by MRI + (PM or DTI) (experimental group) and MRI + NE (control group), and the decompression levels determined by MRI + NE; VAS-BP, VAS-LP, and ODI; measurement variables of patient demographics, comorbidities, surgical details between two groups. *t* test analysis was undertaken to compare decompression levels; VAS-BP, VAS-LP, and ODI; measurement variables of patient demographics, comorbidities, and surgical details between two groups. Chi-square test or Fisher’s exact test was undertaken to compare categorical variables of patient demographics, comorbidities, and surgical details between the experiment and the control group. On the basis of a type I error rate of 5 % and a power of 90 %, we set the target sample size at 100 patients. All statistical analysis was done using IBM SPSS version 19.

## Results

### Characteristics of the study population

From October 2013 through October 2015, a total of 114 patients (55 in the experimental group, 59 in the control group) with degenerative lumbar spinal stenosis detected on MRI were enrolled.

### Outcomes

#### In the experimental group

As shown in Table 1, the reference FA values were taken by L1 levels in 54 patients; while in 1 patient, they were replaced by L2 level because the L1 level was narrow. Averages of the reference FA values and levels were cauda equina, 0.437 ± 0.028 (55); left nerve root, 0.457 ± 0.026 (55); and right nerve root, 0.467 ± 0.026 (55). Averages of the FA values of positive levels and levels were cauda equina, 0.295 ± 0.034 (99); left nerve root, 0.312 ± 0.034 (11); and right nerve root, 0.310 ± 0.038 (18). Averages of the FA values of negative levels and levels were cauda equina, 0.408 ± 0.045 (76); left nerve root, 0.484 ± 0.072 (76); and right nerve root, 0.487 ± 0.055 (76). No statistically significant difference was found between two radiologists about FA values. Averages of the PM scores of positive levels and levels were left nerve root, 3.38 ± 1.35 (55); right nerve root, 3.02 ± 1.29 (51). Averages of the PM scores of negative levels and levels were left nerve root, 1 (25); right nerve root, 1 (29) (Table 2). All lumbar spinal levels were with respect to decompression levels determined only by MRI except for reference levels.

**Table 1:** Averages of FA values of levels and levels

Reference FA values^a^ (n)			Positive FA values(n)			Negative FA values(n)		
Cauda equinas (n)	Left nerve roots (n)	Right nerve roots (n)	Cauda equinas (n)	Left nerve roots (n)	Right nerve roots (n)	Cauda equinas (n)	Left nerve roots (n)	Right nerve roots (n)
0.437 ± 0.028 (55)	0.457 ± 0.026 (55)	0.467 ± 0.026 (55)	0.295 ± 0.034 (99)	0.312 ± 0.034 (11)	0.310 ± 0.038 (18)	0.408 ± 0.045 (76)	0.484 ± 0.072 (76)	0.487 ± 0.055 (76)

^a^All levels were considered to be surgical decompression determined by conventional MRI; ^b^the reference FA values were taken by L1 levels in 54 patients, while in 1 patients, they were replaced by L2 levels because of the L1 levels were narrow; ^c^All the negative FA values were considered only nerve roots and/or cauda equinas in the negative levels, not that in the positive levels;d, If the FA value of lumbar cauda equina or/and the nerve root of the narrow level decreased ≥ 0.1 than the reference FA value, the level was positive; *FA* fractional anisotropy

**Table 2:** Summary of levels and scores of PM

Project	Positive PM scores		Negtive^a^ PM scores	
	Left nerve roots	Right nerve roots	Left nerve roots	Right nerve roots
Levels(n)	55	51	25	29
Averages (scores one level)	3.38 ± 1.35	3.02 ± 1.29	1	1

*PM* paraspinal mapping; ^a^M scores = 1,one level, one side

As shown in Table 3, the FA values of positive levels compared with the reference FA values were decreased with statistically significant differences (cauda equina, *p* = 0.000; the left nerve root, *p* = 0.000; the right nerve root, *p* = 0.000, respectively). The PM scores of positive compared with the negative PM scores were obviously increased with statistically significant differences (left nerve root, *p* = 0.000; right nerve root, *p* = 0.000) (Table 4).

**Table 3:** The p-values by t-test analysis of FA values

Test values	Reference and positive FA values of cauda equina	Reference and positive FA values of left nerve root	Reference and positive FA values of right nerve root
p-values	0.000	0.000	0.000^a^

*FA* fractional anisotropy; ^a^Because equal variances were not assumed, Satterthwaite separate variance estimation t-test was selected

**Table 4:** The p-value of PM scores of nerve roots

Test value	Left(positive and negative ^a^)	Right(positive and negative^a^)
*p*-value	0.000^b^	0.000^b^

*PM* paraspinal mapping; ^a^PM scores = 1,one level, one side; ^b^because equal variances were not assumed, Satterthwaite separate variance estimation t-test was selected

The positive, specificity, and positive and negative predictive value of PM, DTI, and (PM or DTI) in distinguishing which are clinically relevant from the decompression levels determined by MRI were the positive, 74 % (PM), 95 % (DTI), 100 % (PM or DTI); specificity, 100 % (PM), 100 % (DTI), 100 % (PM or DTI); positive predictive value, 100 % (PM), 100 % (DTI), 100 % (PM or DTI); negative predictive value, 69 %(PM), 92 % (DTI), 100 % (PM or DTI) (Table 5). Levels determined by MRI were 184 and that of clinically irrelevant were 67 with its percentage 36 %.

**Table 5:** The positive,specificity, positive and negative predictive value of PM, DTI and (PM or DTI) in distinguishing which are clinically relevant from the decompression levels determined by MRI

Projects	Positive levels n	Negative levels n	Positive rate n%	Specificity n%	Positive predictive value n%	Negative predictive value n%
PM	87	30	74 %	100 %	100 %	69 %
DTI	111	6	95 %	100 %	100 %	92 %
(PM or DTI)	117	0	100 %	100 %	100 %	100 %

*DTI* diffusion tensor imaging, *PM* paraspinal mapping

#### In the experimental and the control group

Between the experiment and control groups, there were differences in demographic, comorbidities, presence of spondylolisthesis and scoliosis, and preoperative ODI, VAS-BP, and VAS-LP scores, but none was statistically significant (Table 6).

**Table 6:** Patient demographics and clinical characteristics

Demographics and surgical characteristics	Experiment group (n = 55)	Control group (n = 59)	*p*-value
Age	61.78 ± 10.05	64.47 ± 9.18	0.138
Sex, n (%)			0.334
Male	23(42 %)	30(51 %)	
Female	32(58 %)	29(49 %)	
BMI (kg/m 2),	35.70 ± 3.47	36.63 ± 4.63	0.226
Comorbidities, n (%)			
Hypertension, n (%)	21(38 %)	26(44 %)	0.524
Coronary artery disease, n(%)	6(11 %)	7(12 %)	0.873
Ulcer, n(%)	2(4 %)	1(2 %)	0.609b
Diabetes mellitus, n (%)	2(4 %)	4(7 %)	0.680b
Cholecystitis, n (%)	2(4 %)	2(3 %)	1.000b
Arrhythmias, n (%)	3(5 %)	1(2 %)	0.351b
Presence of spondylolisthesis, n (%)			
Yes	28(51 %)	27(46 %)	0.583
No			
Scoliosis, n (%)			0.443
Yes	26(47 %)	31(53 %)	0.574
No			
Preoperative back pain (VAS-BP)	99.72 ± 21.76	104.92 ± 18.26	0.170
Preoperative leg pain (VAS-LP)	105.58 ± 18.55	108.86 ± 13.54	0.280
Preoperative ODI	43.78 ± 7.30	43.68 ± 5.84	0.934a
Levels determined by MRI + NE	2.76 ± 0.79	2.64 ± 0.87	0.445

^a^Because equal variances were not assumed, Satterthwaite separate variance estimation t-test was selected; ^b^Fisher’s exact test was selected because of the minimum theoretical expected frequency < 5; *BMI* body mass index, *BP* back pain; *LP* leg pain, *ODI* oswestry disability index, *VAS* visual analog scale, *MRI* magnetic resonance imaging, *NE* neurogenic examination

The levels of decompression determined by MRI + (PM or DTI) in the experimental group were statistically significantly less than that determined by MRI + NE in the control group (*p* = 0.000) (Table 7). However, levels of decompression determined by MRI + NE both did not show differences between the experimental and control groups (*p* = 0.445) (Table 6). No opinion conflict happened in the decision of decompression level with all patients.

**Table 7:** Analysis of the outcome measures

Outcome measure	Experiment group (n = 55)	Control group (n = 59)	p-value
Levels of decompression	2.13 ± 0.64	2.64 ± 0.87	0.000^a^
Operative time (min)	176.73 ± 63.28	215.32 ± 52.07	0.001
Blood loss	376.72 ± 247.90	502.54 ± 270.44	0.011
transfusion, n (%)	17(31 %)	36(61 %)	0.001
Leg dysesthesia, n (%)	3(5 %)	0(0 %)	0.109^b^

^a^Because equal variances were not assumed, Satterthwaite separate variance estimation t-test was selected; ^b^Fisher’s exact test was selected because of the minimum theoretical expected frequency < 5; *BP* back pain, *LP* leg pain, *ODI* oswestry disability index, *VAS* visual analog scale

**Fig. 5:**
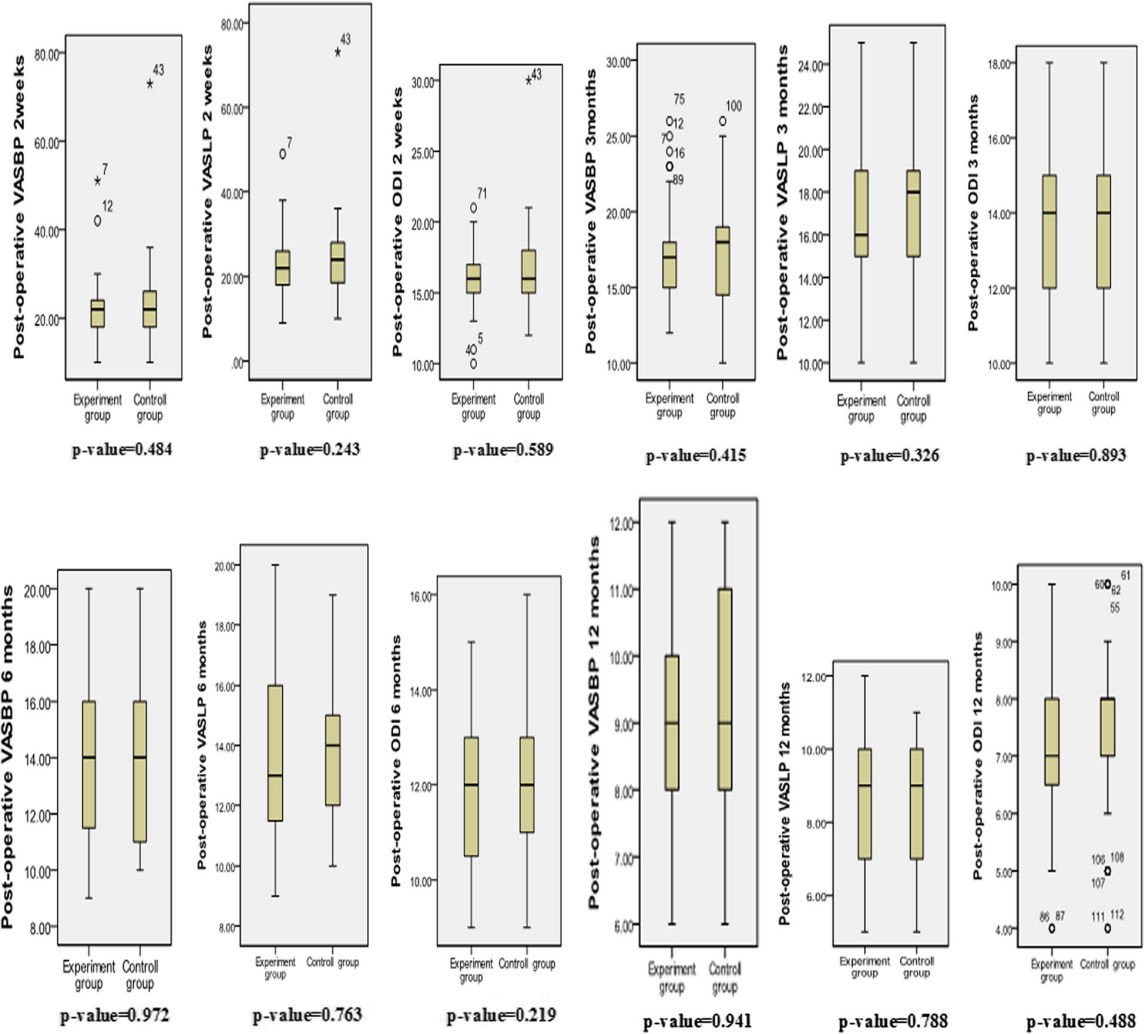
Comparison between two groups of improvements in functional and pain scores at 2 weeks, 3 months, 6 months, and 12 months after surgery. Median values and ranges are presented. *ODI* Oswestry Disability Index, *VASBP* visual analog scale for back pain, *VAS-LP* visual analog scale for leg pain

The surgical time, blood loss, and surgical transfusion were statistically significantly less in the experimental group (*p* = 0.001, *p* = 0.011, *p* = 0.001, respectively). There were no differences in leg dysesthesia complications (*p* = 0.109) (Table 7). There were no differences in improvement of VAS-BP, VAS-LP, and ODI scores 2 weeks, 3 months, 6 months, and 12 months after operation between the experimental and control groups (Fig. 5).

## Discussion

Yamashita et al. [30] have demonstrated the feasibility of whole-body MR neurography with the use of DWI that can depict tissues with an impeded diffusion, such as tumors, brain, spinal cord, and peripheral nerves. MR neurography by using DWI can clearly show lumbar spinal nerves, and the mean ADC in the nerve root entrapment with foraminal stenosis is higher than in the intact nerve roots by using MR imaging at 1.5 T [31]. The ADC map is limited because the tissue contrast between the nerves and surrounding tissues is poor [32]. FA had a much higher sensitivity and specificity (73.3 and 100 %) in the detection of the spinal cord abnormalities compared with T2-weighted FSE imaging (46.7 and 100 %) and ADC (13.4 and 80 %) [33].

A few recent DTI studies of lumbar spinal nerve were demonstrated by Balbi et al. [34] at 1.5 T and van der Jagt et al. [35] and Budzik et al. [36] at 3 T. Also, DTI studies of the cauda equina were demonstrated by Tsuchiya et al. and Filippi et al. [37, 38]. all these showed that DTI can determine the FA of the spinal nerves and/or cauda equina in patients and healthy volunteers.

In this study, averages of reference FA values were cauda equina, 0.437 ± 0.028; left nerve root, 0.457 ± 0.026; and right nerve root, 0.467 ± 0.026. Averages of the FA values of negative levels and levels were cauda equina, 0.408 ± 0.045; left nerve root, 0.484 ± 0.072; and right nerve root, 0.487 ± 0.055. Our FA values of nerve roots were not comparable to those obtained in the study of lumbar spinal nerves by Balbi et al. [34] (0.218), van der Jagt et al. [35] (0.31), and Budzik et al. [36], which might be due to the different software calculation methods. Our reference FA value and negative FA value of cauda equina were larger than the gray matter (0.32 ± 0.11), less than the white matter (0.63 ± 0.08) [39, 40], as well as lower than the average of cauda equina (0.492) [38]; because at the L1 level, the FA value we measured was actually a FA value of mixture of gray matter, white matter, and cerebrospinal fluid; at L2—S1 levels, the FA value we measured was actually a FA value of mixture of cauda equina nerve and cerebrospinal fluid. the cerebrospinal fluid would reduce the FA value.

By contrast, the FA values of positive levels compared with the reference FA value that were decreased with statistically significant differences which showed that reduction of the FA value ≥0.1 than the reference FA value was of statistical significance. Eguchi et al. [27] showed that the mean FA of the proximal nerve roots on the side of entrapment was 0.128 ± 0.036, which is significantly lower than the 0.213 ± 0.042 on the intact side, and the mean FA of the distal lumbar spinal nerve roots on the side of entrapment was 0.131 ± 0.014, significantly lower than the 0.242 ± 0.032 seen on the intact side (*p* ≥ 0.001). The difference between normal side and entrapment side values was about 0.1; our pre-experiment also showed that the FA value of cauda equina and/or nerve roots of the level was ≤0.1 than that of the normal level which was clinically meaningful. According to the schema above, we set the standard as follows: If the FA value of lumbar cauda equina and/or nerve roots of the narrow level decreased ≥0.1 than that of the non-stenotic and normal level (commonly taken T12–L1 cauda equina and nerve root value as reference), it was positive and the level should be treated surgically.

Although the mechanisms of decreasing FA in nerve roots have been controversial, these findings suggest that diffusion in the tissue had become more isotropic because of edema, in which fluid is trapped in the tissue, creating an isotropic environment and a reduction in FA. These hypotheses have been supported by previous experimental studies. Beaulieu et al. [41, 42] reported that Wallerian degeneration after peripheral nerve injury reduces the anisotropy of water diffusion. Several studies indicated that the FA of peripheral nerves was strongly correlated with the axonal degeneration and regeneration in rat and mouse sciatic nerves [43, 44]. The decrease in the FA values may reflect the degree of microstructural disorganization of the spinal cord, suggesting either local extra-cellular edema or a smaller number of fibers matching a larger extracellular space, or both. On the other hand, minor lesions and edema with roughly preserved fibrillary microstructure of the spinal cord are not associated with major FA changes, which opposes to the demyelination, cavitations, and necrotic changes [45]. Thus, the high FA values suggest that the microstructure of the spinal cord is preserved, even in cases with high signal intensity of the spinal cord on T2-weighted images, maybe so does the cauda equina.

Our 3.13 × 2.54 × 3.0 mm^3^ voxel size was larger than that in the previous study (1.1 × 1.6 × 3.0 mm^3^), and therefore spatial resolution was unlikely to account for the difference [38]; it might be due to attempts to increase resolution by decreasing voxel size would lead to a bad result in lumbar nerve root imaging. The FA values of the cauda equina were typically lower than the actual values which might be due in part to volume averaging with cerebrospinal fluid (CSF) in each voxel. All the above affected FA values of the reference and narrow levels but not their difference.

The PM scores of positive compared with the negative PM scores obviously increased with statistically significant differences (left nerve root, *p* = 0.000; right nerve root, *p* = 0.000) which showed that the standard of PM was statistically significant.

Levels of decompression determined by MRI + (PM or DTI) in the experimental group were less, statistically significant than that determined by MRI + NE in the control group which demonstrated that the use of PM and DTI can further prevent the occurrence of false positives with conventional MRI, distinguish which are clinically relevant from the cauda equina and nerve root lesions based on MRI, and determine and reduce the decompression levels of lumbar spinal stenosis than MRI + NE.

A positive EMG, based on spontaneous activity findings, can reassure clinicians that a lesion seen on an imaging study is indeed a pain generator [46]. Haig et al. [47] argued that imaging does not differentiate between symptomatic from asymptomatic individuals, whereas electrodiagnosis does. They believe that the radiographic findings alone are insufficient to justify the treatment for spinal stenosis. In chronic degenerative myelopathy caused by disc herniation or degenerative spinal canal stenosis, significant decrease of FA has been found, including cases with no visible changes in the spinal cord on plain MRI [45, 48, 49]. In recently published reports on the contribution of DTI in cervical myelopathy, the authors have claimed that DTI proved to be more sensitive than conventional T2-weighted images in the assessment of cervical degenerative myelopathy [45, 49–51]. Our results further indicated that DTI or PM can accurately identify the cauda equina and/or nerve root lesions than MRI in lumbar spinal stenosis, aviode the occurences of false positive with MRI.

The positive and the negative predictive values of (PM or DTI) in distinguishing which are clinically relevant from the decompression levels determined by MRI were all 100 % which demonstrated good diagnostic effect.

Because decompression levels in the experimental group were statistically significantly reduced compared with the control group, the corresponding surgical blood loss, surgical time, and surgical transfusion in the experimental group were also statistically significantly reduced than that in the control group. Apparently on reducing the decompression levels, the surgical dissection and complexity of the surgical procedure were reduced, which in turn reduced the amount of bleeding, surgical time, and surgical transfusion. The experimental group reported three cases of leg dysesthesia because of surgical complications, and no such events were reported in the control group; however, there was no statistically significant difference in terms of complications between the two groups.

In the follow-up, the averages of postoperative VAS-BP, VAS-LP, and ODI scores were comparable between the two groups; in the other words, the experimental group not only decreased decompression levels, surgical time, blood loss, and surgical transfusion but also achieved results of operations equal with that of the control group, thus obviously at an advantage. Although the postoperative VAS-BP, VAS-LP, and ODI scores in some cases had some fluctuation, the average was toward improvement and none of the patients’ symptoms recurred or exacerbated and required a repeat surgery, thus the effect of surgical treatment will stand for the test of time. All these also suggested that the use of PM and DTI to determine surgical levels will not miss the level which should be operated.

To our knowledge, this is the first study of the use of (DTI or PM) + MRI to look for decompression levels of patients with lumbar spinal stenosis. If patients who have no concordance between MRI and NE or decompression levels are longer (≥2) according to MRI + NE, in addition to the use of (DTI or PM), it can further determine and reduce decompression levels and avoid an extensive surgery, therefore reducing surgical trauma and hospitalization expenses etc. Ways to look for the responsibility level of surgery in patients with lumbar spinal stenosis already have provocative discography, discography, temporary external fixation, and facet joint blocks or zygapophyseal joint blocks. However, the disadvantages of these procedures are with invasion, low accuracy and complications [52], and no using (DTI or PM).

### Limitations

We acknowledge that our study has some limitations. One is that a small number of subjects were investigated and had limited follow-up. Further studies are needed to investigate whether our findings remain valid in a larger population and longer follow-up. Another, we could not repeat the DTI and PM after surgery because of spinal instrumentation artifacts, such as those from pedicle screw systems (affecting DTI) and surgical scar (affecting PM).

## Conclusions

This study suggested that the use of PM and DTI can further prevent the occurrence of false positives with conventional MRI, distinguish which are clinically relevant from cauda equina and nerve root lesions based on MRI, and reduce the decompression levels and surgical trauma of lumbar spinal stenosis than MRI + NE, as well as ensure surgical effectiveness. MRI + (PM or DTI) showed clear benefits in determining decompression levels of lumbar spinal stenosis than MRI + NE. In patients with lumbar spinal stenosis, the use of PM and DTI techniques reduces decompression levels and increases the safety and benefits of surgery.

### Ethics approval

This study is in accordance with the ethical standards in the 1964 Declaration of Helsinki and relevant regulations of the US Health Insurance Portability and Accountability Act (HIPAA). Prior to data collection, consent to participate was obtained from all the patients involved in the study.
